# Bullying victimization and its associated factors among adolescents in Illu Abba Bor Zone, Southwest Ethiopia: a cross-sectional study

**DOI:** 10.1186/s40359-022-00967-6

**Published:** 2022-11-10

**Authors:** Hunde Tarafa, Yadeta Alemayehu, Tilahun Bete, Debela Tarecha

**Affiliations:** 1 Department of Psychiatry, College of Health Sciences, Mettu University, Mettu, Ethiopia; 2 Department of Psychiatry, College of Health and Medical Sciences, Haramaya University, Harar, Ethiopia

**Keywords:** Bullying victimization, Adolescents, Mettu, Ethiopia

## Abstract

**Background:**

Bullying victimization is a repetitive exposure to negative actions from one or more students over time. Bullying victim adolescents have higher levels of mental health problems, worse mental health outcomes, and lowered social status than non-victim adolescents. Literature on bullying among adolescents in Ethiopia is limited. This study aimed to assess the magnitude of bullying victimization and its associated factors among adolescents.

**Methods:**

A community-based cross-sectional study was carried out among 847 adolescents in Mettu town. A Stratified sampling technique was used to select eligible Study participants from September, 1 to 30, 2020. Linear regression analysis model was used; first bivariate analysis was performed to see the association of each independent variable with bullying victimization. Variables with (*P* < 0.25) in bivariate analysis were entered into a multivariate linear regression model to identify the association of each independent variable with bullying victimization. The statistical significance was considered at *P* value < 0.05.

**Results:**

From the total of 847 adolescents 819 were voluntarily involved in the study giving the response rate of 96.7%. The prevalence of bullying victimization in this study was 30.4%. Being male (β = 1.135, *p* = 0.001), physical abuse (β = 0.622, *p* ≤ 0.001), emotional abuse (β = 0.512, *p* ≤ 0.001), current substance use (β = 1.153, *p* = 0.005), psychological distress (β = 0.406, *p* ≤ 0.001) and having medical illness (β = 3.500, *p* ≤ 0.001) were significantly associated with bullying victimization.

**Conclusions:**

Bullying victimization is quite common among adolescents and has both short- and long-term consequences. Bullying prevention treatments should focus on male adolescents and those who report being bullied. Anti-bullying policies in schools are critical for educating teachers, parents, and students about bullying.

## Introduction

Adolescence is one of the critical transition times in life that comes after childhood period and before adulthood, and is characterized by an enormous pace in growth and change that is next only to that of infancy [1]. About 85% of adolescents in the world are living in developing countries. In Sub-Saharan Africa, adolescents constitute 20–30% of the population. Several countries in Sub-Saharan Africa have a large and increasing adolescent population that exceeds those from other parts of the world [2, 3]. In Ethiopia about one in four people is adolescent [4].

During this stage, one of the serious problems in schools that have an adverse consequence on the psychological wellbeing of adolescents is bullying. Bullying behavior has been defined as deliberate aggressive behavior repeated over a period of time, where there is an imbalance of power between the bullying victim and the perpetrator [5]. Bullying victimization is a repetitive exposure to negative actions from one or more students over time. An adolescent is a bullying victim when another adolescent says unpleasant and nasty things to her or him, kicks, hit, threatened, sent nasty notes, locked inside a room, and when no one ever talks with [6].

Bullying behavior tends to include verbal bullying, physical bullying, relational bullying, and social bullying. Verbal bullying includes threatening behaviors, nasty teasing, and name-calling. On the other hand, Physical bullying is explained as behaviors directed at the victimized individual (physically hurt, stolen or property damaged) [7]. Relational bullying aims to impair an individual’s friendly relationships through exclusion or tries to break up peer relationships [8]. Similarly, social bullying aims to damage a person’s social standing, usually through spreading nasty rumors or lies about the targeted person, activities often carried out by a third party [8, 9].

Literature has shown that bullying between adolescents is associated with poorer social, physical, psychological, and academic outcomes for both the perpetrators and victim adolescents [10, 11]. Bullying victim adolescents have higher levels of mental health problems, worse mental health outcomes, and lowered social status than non-victim adolescents [12]. In addition, bullying victimization has been associated with serious health problems—for instance, recent systematic reviews indicated strong evidence of a causal relationship between bullying victimization and mental illness such as anxiety, depression, poor general health, and suicidal behaviors [13]. It has also been indicated that bullying victimization is significantly associated with psychological distress and reduced levels of psychological wellbeing [14].

Since there is a scarcity of research on bullying victimization in Sub-Saharan African countries, research on adolescents about bullying victimization and its associated factors is critical, because recognizing and addressing young people's mental health needs helps them function better socially, academically, vocationally, and develop into well-adjusted productive adults. In light of this, it is critical in resource-constrained nations to treat adolescents' mental health in order to strengthen and expand evidence-based intervention. Evidence is needed not only to inform policymakers, but also to raise public awareness of teenage mental health challenges in order to organize social support [15].

To the best of our knowledge, this is the first research to assess bullying victimization and its associated factors among adolescents in Ethiopia. Thus, the findings from this research could help in developing a pro-active anti-bullying program for adolescents. It will guide various sectors such as education sectors, health sectors, and justice sectors in developing a country-wide action plan for the reduction of bullying victimization among adolescents.

## Method and materials

### Study setting and period

The study was conducted in Mettu town. Mettu town is the administrative town of Illu Abba Bor zone, which is found in Oromia regional state, Ethiopia. The town is located 600 km far apart from the capital city of Ethiopia, Addis Ababa. The town has three administrative kebeles. According to the data from the town administration, the number of households located in the town was approximately 22,682 and the overall adolescent population of the Mettu town was 21,844 (21.4% of the overall population of Mettu town). The study was carried out from September, 1 to 30, 2020.

### Study design and population

A community-based cross-sectional study was conducted among all sampled adolescents in Mettu town. Individuals who were acutely ill during the data collection period were excluded from the study.

### Sample size and sampling technique

The sample size was calculated using single population proportion formula ${\text{n}} = \left( {Z_{{\frac{\alpha }{2}}} } \right)^{2} \left( {\frac{{p\left( {1 - p} \right)}}{{d^{2} }}} \right)$ by considering; the proportion of bullying victimization as 50% since there are no published studies in the study area, confidence interval of 95%, 5% margin of error, and design effect of 2.0 were used. Then, adding a non-response rate of 10%. Thus, the total sample size required was 847.

For selecting study participants a stratified sampling technique was used. Before data collection, a preliminary survey was carried out to number households containing adolescents in the town. Accordingly, 4250, 4112, and 4191households (HHS) were identified and numbered from Kebele 1, kebele 2, and kebele 3 respectively. Then the calculated sample size was proportionally allocated for the three kebeles based on the number of households containing adolescents in kebeles. Finally, a simple random sampling technique was utilized to select HHs containing adolescents. At the time when more than one eligible adolescents were faced in the select household, a kish table was used to decide which adolescent was interviewed.

### Variables of the study

The dependent variable was bullying victimization. Independent variables included were socio-demographic related variables (age, sex, ethnicity, level of education, family size, parents living status, educational status of the father, educational status of the mother, occupation of the father, occupation of the mother, parental marital status); psycho-social factors (number close friends, social support, parental substance use, and satisfaction on relationship with close friends); health-related factors (family history of mental illness, having known medical illness, and psychological distress); current substance use and childhood trauma history.

### Data collection instruments

#### Forms of bullying scale (FBS)

The FBS is a self-report measure of adolescents' exposure to bullying behavior. FBS has a victimization version and perpetration version. It was measured on a 5-point Likert scale ("This did not happen to me"; "once or twice"; "every few weeks"; "about once a week"; and "several times a week or more"). For the current study, FBS victimization version was used, which encompasses ten items that were used to assess bullying victimization (e.g., “I was teased in nasty ways”, “secrets were told about me to others to hurt me”). The sum of FBS victimization version scores can range from 10 to 50. In this study participants who scored above mean on FBS were considered as having bullying victimization. In the current study, the internal consistency (Cronbach alpha) of FBS was (α = 0.90).

#### Childhood trauma questionnaire (CTQ)

The CTQ is a self-reported instrument that can be used to screen for a history of childhood neglect and abuse. It is appropriate for adolescents [16]. The self-report includes 28 items in which participants are asked to rank the frequency (0- never true to 5- very often true) of abuse and neglect experiences they encountered as children [17].

Childhood trauma questionnaire assesses childhood trauma in five categories: emotional abuse, physical abuse, sexual abuse, emotional neglect, and physical neglect. Responses are graded on a 5-point scale (1 = never true, 2 = rarely true, 3 = sometimes true, 4 = frequently true, 5 = very often true). Each subscale is represented by five questions, with a possible score ranging from 5 to 25 [17]. Childhood trauma questionnaire also has a minimization/denial scale (3 items), that screens for the likelihood of underreporting trauma experiences.

### Oslo 3-items social support scale

Social support was measured by using Oslo social support questionnaires which has a score range from 3 to 14 that was interpreted as 3–8 is poor support, 9–11 is moderate support, and 12–14 is strong support [18].

### Kessler psychological distress scale (K10)

To assess psychological distress, K10, a self-report instrument composed of ten items intended to provide a global assessment of distress based on questions about anxiety and depressive symptoms encountered in the past 30 days, was used. The items are scored using a five-point ordinal scale [19]. Each respondent's overall K10 score was derived by summing all ten elements, and scores ranged from 10 to 50 [19]. In this study, the scores were divided into two categories: those who scored < 20 (absence of psychological distress), and those who scored ≥ 20 (presence of psychological distress) [20].

### Data collection procedures and data quality control

Face-to-face interviews were used to collect the data. The data collection process was supervised by two BSc. Psychiatry nurses and collected by five BSc. Clinical nurses. The questionnaire consisted of structured questions that can be subdivided into five different categories: socio-demographic and family-related characteristics, bullying victimization scale, substance use, childhood trauma history, psychological distress, psycho-social, and health-related factors, and Oslo 3-items social support scale.

The questionnaires were pretested one week before actual data collection at Gore town on 5% (n = 43) of the total sample size that was not included in the main study. Based on the pretest, vague and ambiguous questions were revised and adjusted. Data collectors and supervisors were trained for one day by the principal investigator on the questionnaires, parent consent, maintaining the privacy of adolescents, and infection prevention mechanism related to COVID 19. For eligible participants who were not found on the day of data collection, data collectors have revisited the households three times at different time intervals and counted them as non-response. Data collectors' were supervised daily and the filled questionaries' were checked daily by the supervisor and principal investigator. The questionnaire was developed in English and then translated into the local language Afan Oromo and Amharic and back-translated into English by language experts to ensure its consistency. The Afan Oromo and Amharic versions of the questionnaire were used to collect the data.

### Data processing and analysis

Data were checked for completeness and coded. Data were entered using Epi-data manager version 4.6 and exported to SPSS Version 26.0 for analysis. Descriptive statistics such as frequency, percentage, mean, and standard deviation were computed and presented using tables and charts. The linear regression analysis model was used; first bivariate analysis was done to see the association of each independent variable with bullying victimization. Variables with (*P* < 0.25) in bivariate analysis were entered into a multivariate logistic regression model to identify the association of each independent variable with bullying victimization. The statistical significance was considered at *P* value < 0.05.

## Results

### Socio-demographic variables

From the total of eight hundred forty-seven (847) study participants, eight hundred nineteen (819) were voluntarily involved in the study giving the response rate of 96.7%. Out of these study participants, 420(51.3%) were female and 390(48.7) were males. The age range of the respondents was ranged from 10 to 19 years with a mean of 14.9 (SD = 2.798) year. The modal age group was 15–19 years; this age group accounted for 454 participants. The majority of participants 614 (75%) were Oromo ethnic group, and 309 (37.7%) were Orthodox religious followers. Four hundred twenty-three (51.6%) were primary school students (Table 1).

**Table 1:** Socio-demographic characteristics of adolescents in Mettu town, September 2020

Variables	Frequency (n)	Percentage (%)
Age		
10–14	365	44.6
15–19	454	55.4
Gender		
Male	399	48.7
Female	420	51.3
Ethnicity		
Tigrawi	67	8.2
Oromo	614	75.0
Gurage	18	2.2
Amhara	100	12.2
Others*	20	2.4
Level of education		
Primary education	423	51.6
Secondary education	345	42.2
No formal education	51	6.2
Family size		
≥ 4	632	77.2
< 4	187	22.8
Parents living status		
Both alive	713	87.1
Both not alive	22	2.7
Only one parent alive	84	10.3
Educational level of the father		
No formal education	153	18.7
Primary school	310	37.9
Secondary school and above	356	43.5
Parental marital status		
Married/live together	690	84.2
Divorced/separated/single	73	8.9
Widowed	56	6.8
Educational level of the mother		
No formal education	188	22.9
Primary school	361	44.1
Secondary school and above	270	33.0
Mother occupational status		
Unemployed	47	5.7
Employed	772	94.3
Father occupational status		
Unemployed	46	5.6
Employed	773	94.4

*Wolaita, Silte, and Kaffa

### Substance use history of respondents

The study has shown that 276 (33.7%) of the study participants were using substances currently. Of these, 129(15.8%) of study participants were khat users, 49(6.0%) cigarette users, and 82(10.0%) alcohol users (Table 2).

**Table 2:** Bivariate linear regression of factors associated with bullying victimization among adolescents in Mettu town, September 2020 N = 819

Predictor variables	Unstandardized β coefficient	SE	t-value	*P* value	95%CI	
					Low bound	Upper bound
Gender						
Male	0.769	0.396	1.941	0.053	0.009	1.547
Female (Reference)	1	1	1	1	1	1
Age	0.097	0.071	1.370	0.171	− 0.042	0.236
Level of education						
No formal educational	− 0.254	0.822	− 0.310	0.757	− 1.867	1.359
Primary school	− 0.195	0.397	− 0.491	0.624	− 0.975	0.585
High school and above	0.261	0.402	0.648	0.517	− 0.529	1.050
Family average monthly income	0.528	0.411	1.283	0.200	0.279	1.335
Occupation status of the mother						
Employed(Reference)						
Unemployed	0.377	0.471	0.801	0.424	0.548	1.302
Occupation status of the father						
Employed (Reference)	1	1	1	1	1	1
Unemployed	0.446	0.509	0.876	0.381	0.554	1.446
Number of close friends						
No close friend	0.653	0.288	2.270	0.023	0.088	1.217
≥ 1 (Reference)	1	1	1	1	1	1
Having medical illness						
No(Reference)	1	1	1	1	1	1
Yes	4.256	0.603	7.064	≤ 0.001	3.074	5.439
Family history of mental illness						
No(Reference)	1	1	1	1	1	1
Yes	0.093	0.117	0.791	0.284	− 0.137	0.323
Have your parents used substances in the past 3 months?						
No(Reference)	1	1	1	1	1	1
Yes	0.034	0.444	0.076	0.939	− .839	.906
Current substance use						
No(Reference)	1	1	1	1	1	1
Yes	2.290	.334	6.857	≤ 0.001		1.634
Social support	0.067	0.092	0.728	0.467	− 0.113	0.247
Physical neglect	0.751	0.041	18.15	0.032	0.670	0.832
Emotional neglect	0.086	0.031	2.77	0.026	0.025	0.147
Physical abuse	0.201	0.058	3.44	0.001	0.087	0.316
Emotional abuse	0.313	0.042	7.456	≤ 0.001	0.230	0.395
Sexual abuse	0.092	0.129	0.415	0.210	− 0.107	0.285
Psychological distress	0.419	0.023	17.830	≤ 0.001	0.373	0.465

Dependent variable: bullying victimization

### Childhood trauma related characteristics of respondents

**Fig. 1:**
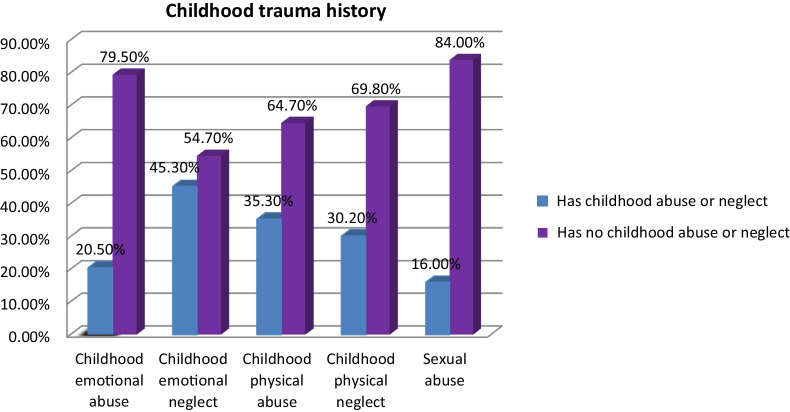
Prevalence of different types of childhood trauma among adolescents in Mettu town, Ethiopia, September 2020, (N = 819)

According to the study, almost one-fifth of the study participants, 168 (20.5%), were emotionally abused, and 371 (45.3%) were emotionally neglected. (Fig. 1).

### Psychosocial factors and health-related characteristics of respondents

Concerning friendship, the majority 726 (88.6%) of the respondents have at least one close friend. The finding of this study shows that 54 (6.6%) of the respondents have family members with a history of mental illness. About one-fourth 201(24.5%) of the respondents had reported that at least one of their parents had used substances in the last three months. More than half 432(52.7%) of the respondents have moderate social support. In this study, 358(43.7%) of the respondents had psychological distress.

### Prevalence of bullying victimization

The prevalence of bullying victimization in this study was 249(30.4%) with 95% CI (26.9, 34.2). Among these 118 (14.4%) were males and 131(16.0%) were females.

### Factors associated with bullying victimization

To identify factors associated with bullying victimization, bivariate and multivariable linear regression analyses were performed. At *p* < 0.25, the bivariate analysis revealed that sex, age, number of close friends, having a medical problem, current substance use history, having a family history of mental illness, physical neglect, emotional neglect, physical abuse, emotional abuse, sexual abuse, and psychological distress and current substance use were all associated with bullying victimization (Table 2).

All the predictor variables with *p* < 0.25 in the bivariate analysis were entered into the multivariable linear regression analysis to identify factors associated with bullying victimization. Male sex, current substance use, emotional abuse, physical abuse, psychological distress, and having a medical illness were found to be significantly associated with bullying victimization in the study (Table 3).

**Table 3:** multivariate linear regression analysis of bullying victimization and its associated factors among adolescents in Mettu town, September, (n = 819)

Predictor variables	Unstandardized β coefficient	SE	t-value	*P* value	95%CI	
					Low bound	Upper bound
Sex						
Male	1.135	0.341	3.332	0.001	0.466	1.803
Female (Reference)						
Current substance use	1.153	0.405	2.844	0.005	0.357	1.948
Physical abuse	0.622	0.049	12.590	≤ 0.001	0.525	0.719
Emotional abuse	0.512	0.040	12.919	≤ 0.001	0.434	0.590
Psychological distress score	0.406	0.019	21.360	≤ 0.001	0.369	0.444
Having medical illness						
Yes	3.500	0.715	4.892	≤ 0.001	2.096	4.904
No (Reference)						

Dependent variable: bullying victimization

For the final model, adjusted R^2^ = 0.664, *P* ≤ .001

Accordingly, Being male increases bullying victimization by 1.135 times than females (β = 1.135, *p* = 0.001). Emotional abuse is significantly and positively associated is bullying victimization (β = 0.512, *p* ≤ 0.001). The interpretation is a one-unit increase in emotional abuse leads to an average of 0.512 unit increase in bullying victimization. The result also showed that physical abuse is significantly and positively associated with bullying victimization (β = 0.622, *p* ≤ 0.001). It means that a one-unit increase in physical abuse results in an average of 0.622 unit increases in bullying victimization.

One unit increase in the psychological distress score results in 0.406 units increases in bullying victimization (β = 0.406, *p* ≤ 0.001). The chance of developing bullying victimization increase by 3.5 times in adolescents with medical illness than in healthy adolescents (β = 3.500, *p* ≤ 0.001). Current substance use increases bullying victimization by 1.153 times that of non-users (β = 1.153, *p* ≤ 0.001).

## Discussion

This study aimed to assess the prevalence and associated factors of bullying victimization among adolescents in Mettu town. The overall prevalence of bullying victimization among adolescents in Mettu town was estimated as 30.4%, 95% CI (26.9, 34.2). This finding is in line with a study conducted in Kuwait [21] where 30.2% of the study participants were found to be the victim of bullying. This may be related to similarities in age range of study participants.

However, this study finding is lower than the findings of studies done in Korea [22], and Egypt [23] with the prevalence rate of 63.4%, and 57.8% respectively. The difference between Korea study and our study is that, in the Korea study a self-administered questionnaire about self-perceptions of bullying victimization was used, whereas in our study interviewer administered questionnaire was used. Moreover, in Korea two questions were used to define the bullying victimization while in the current study Forms of Bullying scale (FBS) was used. Furthermore, the difference might related to sample size in which they have collected data from 2936 participants and whereas our study was conducted on 819 adolescents. Additionally, variation between the Egypt study and our study might also be explained by difference in data collection tool used, which they used short version of aggression and victimization version to assess bullying victimization. In addition the Egypt study used self-administered questionnaire.

Moreover, this finding is much higher than that reported in India [24], North India [25], Korea [26], and Malaysia [27] 15.3%, 25.6%, 8.2%, and16.2% respectively. This discrepancy could be due to the variation in the study population, in which their studies were school based, whereas our study was community based. Moreover, the difference could be due to partially covered adolescence age range (14–19 years) in their studies, while the current study was covered the whole adolescent age range (10–19 years).

Our study found more male adolescents are getting involved in bullying victimization (15%) than female adolescents (11.9%), which is consistent with the findings from other studies. A multi-country study of 40 countries reported that boys’ bullying (from 8.6 to 45.2%) was higher than girls' (4.8–35.8%) [28]. Another cross-sectional study from India shows that boys are more likely to be bullied than girls [29]. The possible explanation for their association might be due to the gender disparities in which boys are more prone to be both bullies and victims of bullying, especially in its physical expression, since girls are more likely to engage in situations of indirect bullying, such as teasing or gossip about peers [30].

In this study, both childhood emotional abuse and physical abuse were significantly associated with bullying victimization among adolescents. This is consistent with studies done in Lebanese [31] and China [32]. Emotional abuse generates parental attachment problems and communication difficulty with colleagues [33]. Likewise, childhood physical neglect escalates the risk of bullying victimization. Childhood parental abuse has a detrimental effect on adolescent-parent relationships and distorts victims' perceptions of stressful situations [34]. Individuals who have been subjected to childhood parental abuse also experience sentiments of disgrace and suffer from interpersonal difficulties including being bullied by others [34, 35].

According to the findings of this study, bullying victimization in adolescents was significantly associated with higher rates of psychological distress. This is supported by research from Mekele High School [20] and Norway [36]. Bullying victimization is widely associated with a mental health problem in public debate, presuming a causal relationship between being bullied and becoming distressed. Losses, abuse, and persistent conflicts or frustrations may moderate or mediate the onset and recurrence of mental health problems, and traumatic events, such as victimization to violence, predispose children and adolescents to mental health problems [37, 38]. Bullying victimization is likely to reflect abuse, conflict, and frustration. Trauma associated with peer interactions, such as being bullied, can create a trauma severe enough to contribute to psychological distress, especially during adolescent growth, when peer relationships are of the biggest importance [39]. Following bullying victimization, increased emotional dysregulation and reduced self-esteem may act as mediators between being bullied and mental health problem such as psychological distress [40]. This could imply that prior mental problems moderate the link between psychological distress and victimization. On the other side, mental health problems may distort the processing of social information: a distressed adolescent with negative self-perception may expect others to respond in a rejective or hostile manner and experience this in social encounters that others intend to be neutral or even positive [41, 42]. Finally, it is likely that adolescents with psychological distress development of social skills and ability to defend themselves are hampered, making them easy targets for bullies. Adolescent mental health problem is known to affect social skills. Bullying victims have been described as submissive and powerless, less popular among peers, and having low self-esteem, all of which may predispose individuals to victimization but may also be precursors to psychological distress [40–42].

The finding of this study showed that having a medical illness is associated with bullying victimization. This finding is consistent with a study done in Kuwait [21]. Peers may perceive them as different due to disease symptoms or treatment regimens. Children with facial disfigurement, for example, may not meet their peer group's beauty standards. Furthermore, children with physical illnesses may be perceived as physically weaker, making them susceptible to bullying victimization [43, 44]. Following that, young people with chronic illnesses are more likely to have poor social functioning (social and communication skills) and academic performance, which may elicit negative reactions from their peers [45].

Our finding showed a significant association between the current substance use and bullying victimization. This finding is consistent with previous studies [27, 46]. Bullying can cause significant physical, social, psychological, and emotional discomfort in adolescents. Bullying victims frequently acquire progressive behavioral illnesses (e.g., depression and anxiety) as a result of being harassed. When combined with a victim's low self-esteem, these circumstances may lead to substance use as a means to cope with how helpless being bullied makes them feel [47].

The following are some of the study's potential limitations that should be noted when interpreting the results: The study's cross-sectional design limits the ability to conclude causality or relationship directions. Some of the tools employed in this study required historical recollection, which could lead to recall bias. Underreporting of sensitive issues such as emotional abuse, physical abuse, physical neglect and emotional neglect within the family, and sexual abuse is possible. We attempted to mitigate this by training interviewers to explain the purpose of the study to participants, interviewing them in an isolated area to protect their privacy, and informing them that their response was anonymous. Some characteristics, such as family history of mental illness and substance abuse, were examined solely through self-report.

## Conclusion

Bully victimization is common among adolescents and is associated with male gender, current substance use, physical abuse, emotional abuse, having a medical illness, and psychological distress. The result may suggest school health programs should focus on those at risk of bullying victimization. Moreover, Bullying prevention treatments should focus on male adolescents and those who report being bullied. Anti-bullying policies in schools are critical for educating teachers, parents, and students about bullying.

## Data Availability

The datasets used and analyzed during the current study are available from the corresponding author on reasonable request.

## Abbreviations

BSc.: Bachelor of sciences
CTQ: Childhood trauma questionnaire
FBS: Forms of bullying scale
K10: Kessler psychological distress scale 10
SPSS: Statistical package for social sciences
USA: United State America
WHO: World Health Organization
